# ARRB1 ameliorates liver ischaemia/reperfusion injury via antagonizing TRAF6‐mediated Lysine 6‐linked polyubiquitination of ASK1 in hepatocytes

**DOI:** 10.1111/jcmm.15412

**Published:** 2020-05-23

**Authors:** Xiaoliang Xu, Zechuan Zhang, Yijun Lu, Qikai Sun, Yang Liu, Qiaoyu Liu, Wenfang Tian, Yin Yin, Hailong Yu, Beicheng Sun

**Affiliations:** ^1^ School of Medicine Southeast University Nanjing China; ^2^ Department of Hepatobiliary Surgery The Affiliated Drum Tower Hospital of Nanjing University Medical School Nanjing China

**Keywords:** ARRB1, ASK1, liver ischemia/reperfusion injury, Lysine 6‐linked polyubiquitination, TRAF6

## Abstract

Hepatic ischaemia/reperfusion (I/R) injury is a major clinical problem during liver surgical procedures, which usually lead to early transplantation failure and higher organ rejection rate, and current effective therapeutic strategies are still limited. Therefore, in‐depth exploring of the molecular mechanisms underlying liver I/R injury is key to the development of new therapeutic methods. β‐arrestins are multifunctional proteins serving as important signalling scaffolds in numerous physiopathological processes, including liver‐specific diseases. However, the role and underlying mechanism of β‐arrestins in hepatic I/R injury remain largely unknown. Here, we showed that only ARRB1, but not ARRB2, was down‐regulated during liver I/R injury. Hepatocyte‐specific overexpression of ARRB1 significantly ameliorated liver damage, as demonstrated by decreases in serum aminotransferases, hepatocellular necrosis and apoptosis, infiltrating inflammatory cells and secretion of pro‐inflammatory cytokines relative to control mice, whereas experiments with ARRB1 knockout mice gotten opposite effects. Mechanistically, ARRB1 directly interacts with ASK1 in hepatocytes and inhibits its TRAF6‐mediated Lysine 6‐linked polyubiquitination, which then prevents the activation of ASK1 and its downstream signalling pathway during hepatic I/R injury. In addition, inhibition of ASK1 remarkably abolished the disruptive effect result from ARRB1 deficiency in liver I/R injury in vivo, indicating that ASK1 was required for ARRB1 function in hepatic I/R injury. In conclusion, we proposed that ARRB1 is a novel protective regulator during liver I/R injury, and modulation of the regulatory axis between ARRB1 and ASK1 could be a novel therapeutic strategy to prevent this pathological process.

## 1 INTRODUCTION

Hepatic ischaemia‐reperfusion (I/R) injury is one of the sources of morbidity and mortality during hepatic resection, liver transplantation, haemorrhagic shock and myocardial infarction.^1^ Tissue injury resulting from I/R is an unavoidable process, which accounts for up to 10% of early transplantation failure and increases the risk of organ rejection and liver dysfunction.^2^ Great efforts have been made to explore the interventions for the treatment of liver I/R injury; however, in clinical practice, only preconditioning is a promising strategy with limited scope of application and beneficial effects.^3^, ^4^ Numerous studies have revealed that hepatic I/R injury is a dynamic process which consists of two processes: ischaemic cellular injury stage and explosive hepatic damage during reperfusion stage, during which extensive cell death and hypernomic inflammatory response are the major features and main culprits of destructive tissue damage.^5^, ^6^ Thus, seeking factors that are simultaneously impeding cell death and sterile inflammation of hepatic I/R injury are critical important for alleviating I/R‐induced liver damage. Several molecular events, such as NF‐κB and mitogen‐activated protein kinase (MAPK) signalling, are activated in hepatic I/R injury, which have multiple effects in the process of cell death and inflammation.^7^, ^8^ Therefore, new effective pharmacological interventions for the treatment of hepatic I/R injury may be identified by targeting these critical regulators.

β‐arrestins are a family of cytoplasmic adaptor proteins consisting of two members: ARRB1 and ARRB2. As their names imply, they have traditionally been identified as terminators of G protein‐coupled receptor (GPCR) signalling transduction and regulatory factors of receptor desensitization upon stimulation.^9^, ^10^ Moreover, emerging observations reveal that they could regulate diverse signalling pathways by acting as essential adaptors for many other protein complexes and thus regulating a wide range of biological processes. Accumulating evidence has confirmed their importance and multiple functions in different human disease. For instance, in brain, ARRB2 regulates Aβ generation and γ‐secretase activity in Alzheimer disease,^11^ and overexpression or activation of ARRB1, but not ARRB2, exerts neuroprotection through coordination of BECN‐1‐dependent autophagy in cerebral ischaemia.^12^ In heart, the expression of ARRB2, but not ARRB1, is up‐regulated in cardiac ischaemia‐reperfusion injury, which then induces cardiomyocyte death by interacting with the p85 subunit of PI3K and negatively regulates the formation of p85‐PI3K/CaV3 survival complex.^13^ But ARRB1 deficiency attenuates rather than exaggerating the cardiomyocyte death and cardiomyopathy induced by myocardial infarction.^14^ In liver, ARRB1 could enhance hepatocellular carcinogenesis through inflammation‐mediated AKT signalling^15^; in addition, ARRB1 could modulate the functions of autoimmune T cells in primary biliary cirrhosis patients.^16^ Also, our recently study revealed that ARRB1 exerts protective function in the pathology of non‐alcoholic steatohepatitis (NASH) by promoting GDF15 maturation,^17^ whereas the expression and function of ARRB family members in the process of hepatic I/R injury still remain unknown. Drawing from these studies, we aimed to determine whether and how ARRB1 and ARRB2 are involved in hepatic I/R injury.

In our preliminary study, we found that ARRB1, but not ARRB2, was significantly decreased in mouse livers subjected to hepatic I/R operation, indicating a potential effect of ARRB1 in this process. Our in vivo and in vitro experiments demonstrated that overexpression of ARRB1 attenuates hepatic I/R injury by inhibiting cell death and ameliorating inflammation, whereas ARRB1 inhibition by genetic deletion exaggerated hepatic I/R injury. Moreover, ASK1 was identified as the vital target of ARRB1, and inhibition of ASK1 obviously recused liver damage resulted from loss of ARRB1 during hepatic I/R injury. Our study revealed and highlighted the vital role of ARRB1‐ASK1 axis in hepatic I/R injury.

## 2 MATERIALS AND METHODS

### 2.1 Animals

Male Pathogen‐free C57BL/6 mice (wide type [WT]) were obtained from Animal Research Center of Nanjing Medical University. ARRB1 KO mice were a kind gift from Dr Bin Wei (Shanghai University). Hepatocyte‐specific overexpression of ARRB1 (Ad‐ARRB1) were created by intravenously injecting adeno‐associated virus (AAV) specifically targeting hepatocyte and expressing ARRB1 or the control virus (Ad‐GFP). Hepatocyte‐specific overexpression of ARRB1 was verified by Western blot (Figure 2A). Animal protocols were approved by the Animal Care and Use Committee of Nanjing Medical University, and experiments were conducted in adherence to the *Guide for the Care and Use of Laboratory Animals* (National Institutes of Health).

### 2.2 Mice hepatic I/R injury model

Mice partial liver ischaemia surgery was conducted as previously described.^7^ Mice were free to access to food and water for 12 hours before surgery. After 60 minutes of liver ischaemia by vascular occlusion, mice were reperfused and killed immediately (0 hours), 2, 6, 12 or 24 hours post‐reperfusion. Sham controls underwent the same operative procedure without vascular occlusion. Serum and tissue samples were collected for further analyses. We intraperitoneally administered the specific ASK1 inhibitor NQDI‐1 (Sigma; 10 mg/kg) to ARRB1 KO and WT mice 30 minutes before the ischaemic surgery. The same volume of DMSO was used as control.

### 2.3 Liver damage and cytokines assessment

Serum levels of alanine aminotransferase (ALT) and aspartate aminotransferase (AST) were measured using an automated chemical analyser (AU5400; Olympus). The extent of parenchymal necrosis in the ischaemic lobes was evaluated using H&E‐stained histological sections and used to calculate the necrotic area as previously described.^18^ The histological severity of hepatic I/R injury was graded using Suzuki's criteria. Serum and medium cytokines TNF‐a, IL‐1β and IL‐6 were detected by ELISA kits (Thermo Fisher Scientific) according to the manufacturer's instructions.

### 2.4 Quantitative real‐time PCR

Total mRNA was isolated from the tissues or cells by TRIzol reagent (Invitrogen) and was reverse‐transcribed with a high‐capacity cDNA reverse transcription kit (TAKARA) according to the manufacturer's instructions. The mRNA levels were quantified by quantitative PCR with SYBR Green (Vazyme). The relative mRNA levels were normalized against GAPDH mRNA levels. The primers used for real‐time PCR are provided in Table S1.

### 2.5 Western blot analysis

Western blot analysis was conducted to measure the levels of proteins in mouse liver tissues or cells as previously described.^7^ Image Lab software was used to quantify the protein expression, and GAPDH was served as the control. Antibodies used for Western blot analyses are presented in Table S2.

### 2.6 Ubiquitination assays

For the endogenous K6‐linked polyubiquitination assay, protein samples were separated under non‐reducing conditions after an IP assay with the indicated antibodies and blotted onto a PVDF membrane (IPVH00010; Millipore). After blocking, the membrane was firstly incubated with anti‐diUbiquitin K6 Affimer reagent with GFP and 7× His tags (AVA00102; Avacta) at 4°C overnight and then with an anti‐His antibody for 1 hour at room temperature, followed by secondary HRP‐conjugated antibodies incubation and protein signals detection.

### 2.7 TUNEL assay

Cell apoptosis was assessed using the In‐Situ Cell Death Detection Kit (11684795910; Roche) following the manufacturer's instructions. Images of TUNEL‐positive cells were captured by fluorescence microscope (DMi8; Leica Microsystems).

### 2.8 Isolation of primary cells

Mouse primary hepatocytes were isolated from 6‐ to 8‐week‐old male mice using a two‐step collagenase perfusion procedure as previously described.^7^ Mouse Kupffer cells were obtained by a further differential centrifugation using Percoll (17‐0891‐02; GE Healthcare Life Sciences) at the same time of isolating hepatocytes, with slight modifications. Briefly, livers were perfused with collagenase buffers and viable cells were then isolated by spinning through 70‐µm mesh (352350; BD Falcon). The hepatocyte fraction was discarded after centrifugation at 50 *g* for 3 minutes and repeated for three times. The supernatant containing non‐parenchymal cells (NPCs) was further centrifuged at 500 *g* for 5 minutes, resuspended in DMEM and carefully layered on the top of Percoll gradients composed of 25% and 50% Percoll layers. After centrifugation at 1500 *g* for 20 minutes, the Kupffer cell fraction was isolated from between the two different Percoll gradient layers.

### 2.9 Hypoxia/reoxygenation (H/R)

To establish the in vitro hepatocyte hypoxia‐reoxygenation (H/R) model, the cells were cultured in serum‐ and glucose‐free DMEM and challenged to a hypoxia state (1% O_2_, 5% CO_2_, and 94% N_2_) in a modular incubator chamber. After 60 minutes of hypoxia, cells were cultured in normoxia situation (air/5% CO_2_) with standard DMEM for the indicated time. And then, the cells and medium were collected for further analysis. Lactate dehydrogenase (LDH) Assay Kit (Promega) was used to determine cell cytotoxicity.

### 2.10 Coimmunoprecipitation

The Coimmunoprecipitation Kit (Thermo Fisher Scientific) was used to detect protein binding. Briefly, L02 cells were cotransfected with Flag‐ARRB1 and His‐ASK1 or His‐ASK1 and HA‐TRAF6 for 2 days and sonicated in ice‐cold immunoprecipitation lysis buffer (Thermo Fisher Scientific). After incubating on ice for 5 minutes with periodic mixing and centrifugation at 13 000 *g* for 15 minutes, the lysates were incubated with antibody‐bound beads for 2 hours at room temperature or overnight at 4°C. Subsequently, the beads were washed twice with immunoprecipitation buffer and once with ultrapure water. Finally, the cell lysates and immune complex were analysed by Western blotting using the indicated primary antibodies.

### 2.11 Flow cytometry

Non‐parenchymal cells (NPCs) were isolated from whole livers (sham) or ischaemic lobes (I/R) following 12 hours of reperfusion. Cells were stained with antimouse F4/80 antibody (BioLegend), Ly6G (BioLegend) and CD11b (BioLegend) to detect neutrophils and macrophages infiltration according to the manufacturer's protocol. We defined Kupffer cells as CD11b^+^F4/80^+^ and neutrophils as CD11b^+^Ly6G^+^. After 30 minutes incubation at 37°C in the dark, suspensions were washed with PBS and suspended with 300 μL PBS. Samples were determined and sorted by FAC‐SCanto^™^ II flow cytometer (BD Biosciences), and the data were analysed with Flow Jo (Tree Star).

### 2.12 Immunohistochemical assay

Sections were deparaffinized followed by rehydration steps through a graded ethanol series and distilled water and treated with 3% H_2_O_2_ in methanol for 30 minutes to block endogenous peroxidase activity. Sections were rinsed twice in phosphate buffered saline for 5 minutes each time and incubated with 10% normal goat serum for 30 minutes to block non‐specific antibody binding. After washing, samples were incubated with primary anti‐rabbit ARRB1 (ab32099) and anti‐goat ARRB2 (ab31294) antibodies at 4°C overnight, washed in phosphate buffered saline three times and incubated with the appropriate secondary antibodies. Sections were subsequently stained with diaminobenzidine (DAB) according to the manufacturer's protocols and mounted and photographed using a digitalized microscope camera (Nikon).

### 2.13 Statistical analysis

All data were analysed with Student's t test or ANOVA followed by post hoc tests. Data are presented as mean ± SD unless stated. *P* < .05 was considered significant. Statistical analysis was performed in GraphPad Prism version 7.0. The statistical methods used and the corresponding *P* values for the data shown in each figure panel are included in the figure legends.

## 3 RESULTS

### 3.1 Hepatic ARRB1 expression level is decreased during hepatic I/R injury

**FIGURE 1:**
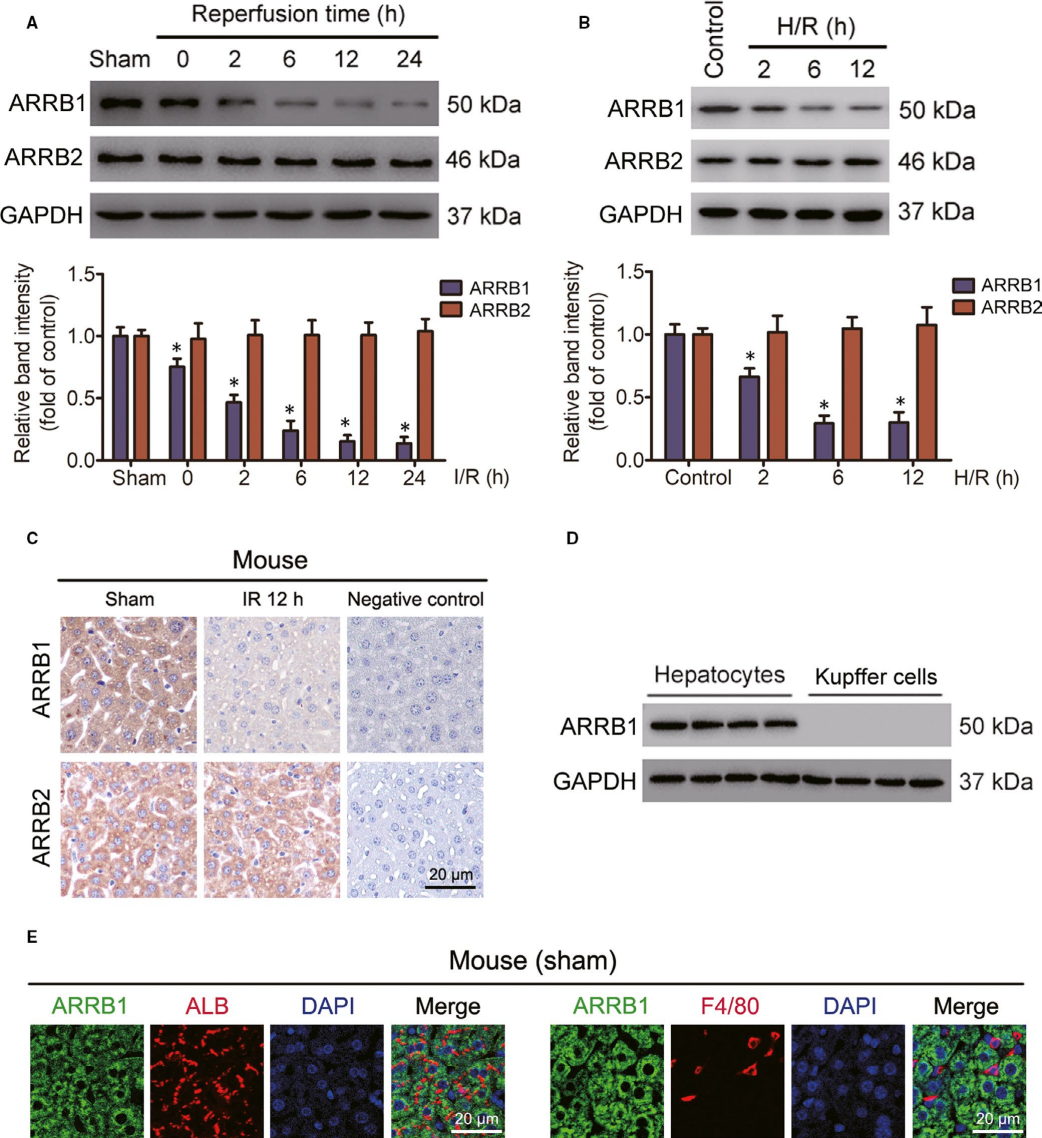
ARRB1 down‐regulation is associated with hepatic I/R injury. A, Western blot analysis of the protein expression of ARRB1 and ARRB2 in livers from mice subjected to I/R operation for indicated time or sham treatment. GAPDH served as the internal control (n = 5‐6 per group, *P < .05 compared with sham group). B, The protein expression levels of ARRB1 and ARRB2 in cultured primary hepatocytes treated with H/R challenge or not (representative of three independent experiments, *P < .05 compared with the control group). GAPDH served as the internal control. C, Representative images of immunohistochemistry detection showing ARRB1 and ARRB2 in the liver sections of mice after hepatic ischaemia for 60 min and at 12 h after reperfusion (magnification, ×400). D, The expression level of ARRB1 in mouse liver hepatocytes and Kupffer cells was detected by Western blot (n = 4 per group). E, Representative immunofluorescence staining showing the expression of ARRB1 (green) and ALB (red) in hepatocytes (left panel), ARRB1 (green) and F4/80 (red) in Kupffer cells (right panel) in mouse liver sections (magnification, ×400). All data are presented as the mean ± SD; significance determined by Student's two‐tailed *t* test

To explore whether β‐arrestins exert functions in the process of hepatic I/R injury, we firstly detected the expression levels of ARRB1 and ARRB2 in liver tissues from WT mice challenged with hepatic I/R surgery and found that both the protein and mRNA levels of ARRB1, but not ARRB2, gradually decreased during the 60‐min portal vein occlusion and subsequent reperfusion process (Figure 1A and Figure S1A). Next, primary hepatocytes from WT mice were isolated and cultured, and then subjected to 1 hour of hypoxia, followed by reoxygenation stimulation for different time courses, and both the protein and mRNA expressions of ARRB1, but not ARRB2, decreased compared with control hepatocytes (Figure 1B and Figure S1B). Furthermore, immunohistochemical staining assays also verified the down‐regulation of ARRB1, but not ARRB2, in the liver lobes from mice subjected to hepatic I/R insult (Figure 1C). Studies have revealed that both hepatocytes and Kupffer cells exert important functions during the process of hepatic I/R injury. Considering the vital function of Kupffer cell during hepatic I/R injury and to further accurately ascertain the function of ARRB1 in the development of liver I/R injury, we also isolated Kupffer cells for further study. Followed RT‐qPCR, Western blot and immunofluorescence staining analyses revealed that ARRB1 was mainly expressed in hepatocytes, but not in Kupffer cells (Figure 1D‐E and Figure S1C). Collectively, our results indicate that ARRB1 down‐regulation in hepatocytes may be associated with hepatic I/R injury and may exert important function in this process.

### 3.2 ARRB1 protects against liver damage induced by hepatic I/R injury

**FIGURE 2:**
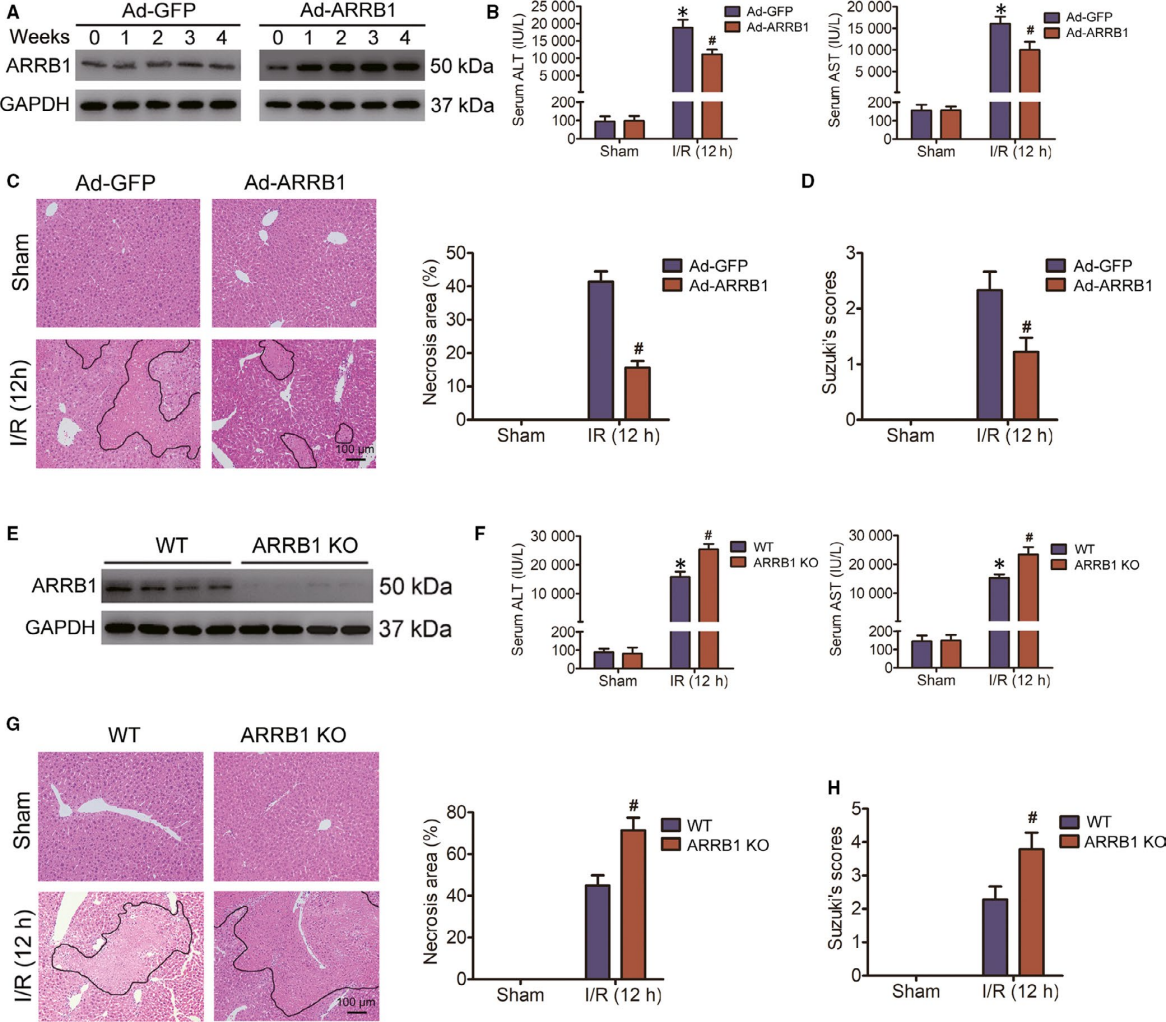
ARRB1 protects against hepatic I/R injury. A, After virus injection, primary hepatocytes isolated at the indicated time‐points were subjected to Western blot to detect the transfection efficiency. B, Serum AST and ALT activities in Ad‐GFP or Ad‐ARRB1 mice subjected to sham or hepatic I/R for 12 h of reperfusion as indicated (n = 5‐6 per group). C, Representative images of histological H&E staining showing necrotic areas in livers from Ad‐GFP or Ad‐ARRB1 mice in the sham and I/R group at 12 h following reperfusion (magnification, ×200, n = 5‐6 per group). D, The Suzuki's scores of liver sections in C. E, Western blot analysis of ARRB1 in primary hepatocytes from WT and ARRB1 KO mice (n = 4 per group). F, The activities of AST and ALT in the serum of WT and ARRB1 KO mice challenged with sham or hepatic I/R at 12 h of reperfusion (n = 5‐6 per group). G, Representative H&E staining images, statistics showing necrotic areas of liver lobes from WT and ARRB1 KO mice in the sham and I/R group at 12 h following reperfusion (magnification, ×200, n = 5‐6 per group). H, The Suzuki's scores of liver sections in G. For B‐D, *P < .05 compared with sham group; ^#^ *P* < .05 compared with corresponding Ad‐GFP group. For F‐H, *P < .05 compared with sham group; ^#^ *P* < .05 compared with corresponding WT group. All data are presented as the mean ± SD; For C, D and G, H, significance was determined by Student's two‐tailed *t* test; For B and F, significance was determined by one‐way ANOVA

To assess the function of ARRB1 in liver I/R injury, we constructed mice with hepatocyte‐specific ARRB1 overexpression by injecting C57BL/6 mice intravenously with adeno‐associated virus expressing ARRB1 (Ad‐ARRB1) or the control virus (Ad‐GFP). To determine the overexpression efficiency of ARRB1, primary hepatocytes were extracted at various time‐points after virus injection, and followed Western blot analysis indicated that ARRB1 overexpression peaked 2 weeks after virus injection and then maintained its level over the next few weeks (Figure 2A). Virus injection itself did not cause liver damage (data not shown). Considering the transfection efficiency of AAV‐mediated ARRB1 overexpression in liver, hepatic I/R surgery was conducted 2 weeks after virus administration. According to the levels of the serum aminotransferases AST and ALT, the degree of liver injury was evaluated. As shown in Figure 2B, liver I/R surgery caused liver damage in both Ad‐GFP and Ad‐ARRB1 mice; in addition, compared with Ad‐GFP controls, ARRB1 overexpression dramatically attenuated AST and ALT activities. According to the histological analysis of livers, we gotten less‐severe hepatic I/R damage in Ad‐ARRB1 mice than that in Ad‐GFP mice after liver I/R challenge, which shown decreased necrotic area and Suzuki's score (Figure 2C‐D).

To further verify the protective effect of ARRB1 in liver I/R injury, we also generated ARRB1 KO mice and subjected them to liver I/R surgery, and the deletion efficiency of ARRB1 in hepatocytes was confirmed by Western blot (Figure 2E). After 60 minutes of ischaemia followed by 12‐hour reperfusion, the activities of serum AST and ALT were significantly increased in ARRB1 KO mice compared with that in WT group (Figure 2F). Histological examination indicated that knockout of ARRB1 obviously enhanced liver damage during hepatic I/R injury, which was visualized with increased necrotic area and Suzuki's score (Figure 2G‐H). Taken together, our results indicate that ARRB1 could exert a protective function during hepatic I/R injury.

### 3.3 ARRB1 inhibits hepatocytes cell death during hepatic I/R injury

**FIGURE 3:**
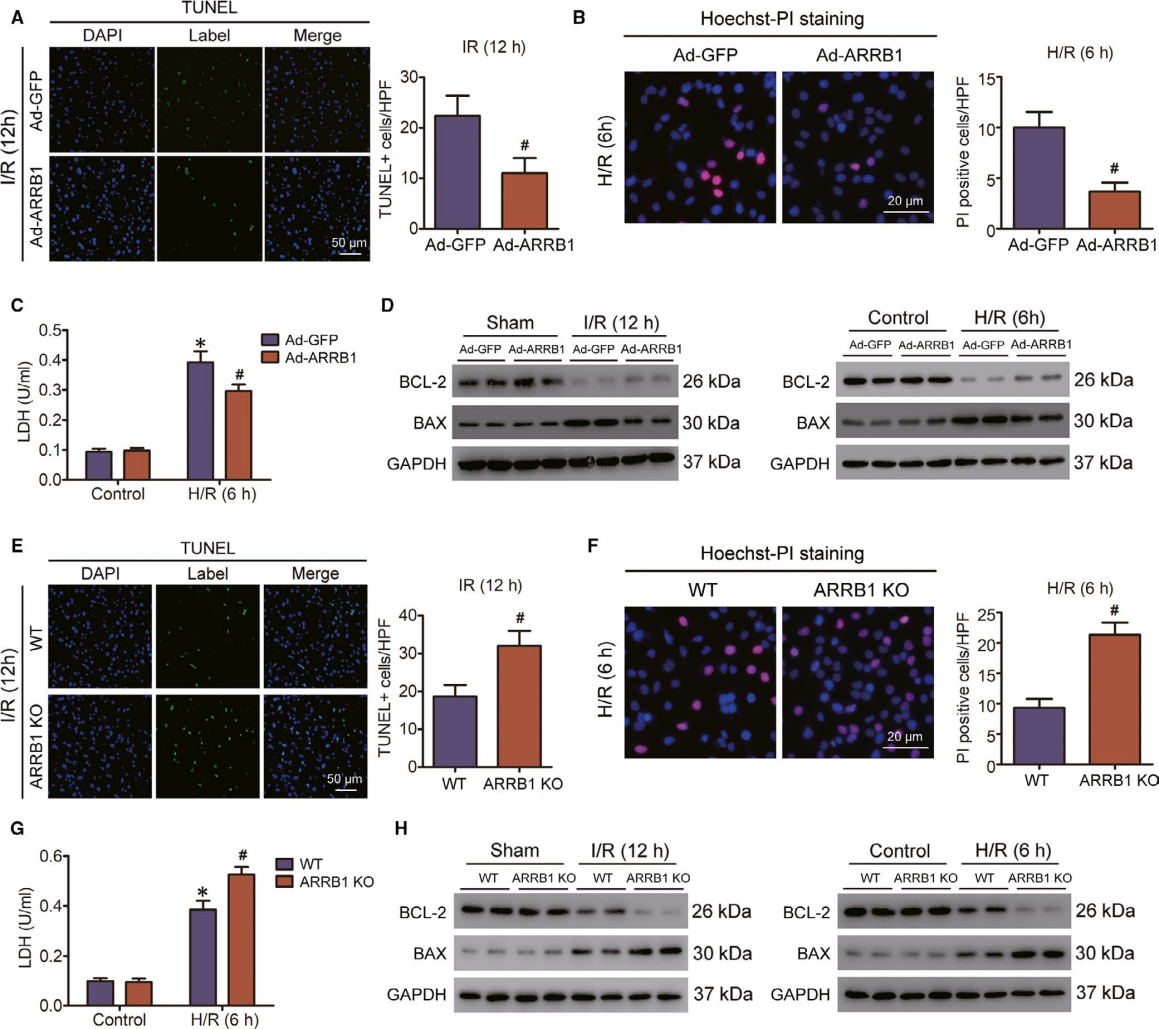
ARRB1 inhibits hepatocyte death during hepatic I/R injury. A, Representative TUNEL immunofluorescent staining in liver lobes of Ad‐GFP and Ad‐ARRB1 mice 12 h following reperfusion (magnification, ×200, n = 5‐6 per group). B, Representative images of Hoechst (blue) and PI (red) staining on primary hepatocytes after hypoxia‐reoxygenation challenge (n = 3 independent experiments). C, LDH release assay of primary hepatocytes from Ad‐GFP and Ad‐ARRB1 subjected to hepatic H/R at 6 h of reoxygenation and their littermate control (n = 3 independent experiments). D, Protein levels of cell death‐related factors in liver lobes from the indicated groups at 12 h after I/R insult and in the primary hepatocytes of different groups at 6 h post‐H/R treatment. GAPDH served as the loading control. E, Representative TUNEL immunofluorescent staining in liver lobes of WT and ARRB1 KO mice 12 h following reperfusion (magnification, ×200, n = 5‐6 per group). F, Representative images of Hoechst (blue) and PI (red) staining on primary hepatocytes after hypoxia‐reoxygenation challenge (n = 3 independent experiments). G, LDH release assay of primary hepatocytes from WT and ARRB1 KO subjected to hepatic H/R at 6 h of reoxygenation and their littermate control (n = 3 independent experiments). H, Protein levels of cell death‐related factors in liver lobes from the indicated groups at 12 h after I/R insult and in the primary hepatocytes of different groups at 6 h post‐H/R treatment. GAPDH served as the loading control. For A‐C, *P < .05 compared with sham or control groups; ^#^ *P* < .05 compared with corresponding Ad‐GFP group. For E‐G, *P < .05 compared with sham or control groups; ^#^ *P* < .05 compared with corresponding WT group. All data are presented as the mean ± SD; for A, B and E, F, significance was determined by Student's two‐tailed *t* test; For C and G, significance was determined by one‐way ANOVA

As is known to all, cell death during hepatic I/R injury is a significant feature of and a direct cause of liver injury.^19^ To investigate whether the blocking of hepatic I/R injury induced by ARRB1 overexpression in liver is mediated by restraining cell death, TUNEL assays were conducted on the liver tissues from mice after I/R challenge. As expected, there were less TUNEL‐positive cells in the liver lobes of Ad‐ARRB1 mice compared with Ad‐GFP mice at 12‐hour after reperfusion, indicating an inhibitory function of ARRB1 on hepatocytes cell death (Figure 3A). In addition, primary hepatocytes of Ad‐ARRB1 and Ad‐GFP mice were isolated and then subjected to hypoxia/reoxygenation (H/R) insult. And the inhibitory effect of ARRB1 on hepatocyte cell death was also validated by Hoechst‐PI staining assays (Figure 3B). H/R challenge of primary hepatocytes caused LDH release and then worsens cell death; here, we found that Ad‐ARRB1 overexpression in primary hepatocytes attenuated the release of LDH (Figure 3C). In accordance with the results, ARRB1 overexpression further increased the expression of the pro‐survival gene BCL‐2 and caused a decrease in the expression of pro‐apoptotic gene BAX at both protein and mRNA levels in the liver tissues after hepatic I/R challenge and in primary hepatocytes after H/R insult (Figure 3D and Figure S2A,C).

In addition, we also investigate the difference of hepatocytes cell death during I/R injury upon ARRB1 deletion in vivo and in vitro. TUNEL‐staining assays revealed that the mount of TUNEL‐positive cells was obviously increased in the liver sections of ARRB1 KO mice compared with the controls (Figure 3E). Also, this phenomenon was confirmed in hepatocytes upon H/R insult by Hoechst‐PI staining assays (Figure 3F). LDH release assays certified that hepatocyte H/R insult indeed caused hepatocyte damage, and ARRB1 deletion obviously enhanced this phenomenon (Figure 3G). Also, the protein and mRNA levels of pro‐survival gene BCL‐2 were decreased, whereas the levels of pro‐apoptotic gene BAX were increased upon ARRB1 deletion during hepatic I/R insult or hepatocytes H/R challenge (Figure 3H and Figure S2B,D). Taken together, our data indicate that ARRB1 could exert an inhibitory effect on hepatocytes cell death during liver I/R injury.

### 3.4 ARRB1 restrains inflammatory response in hepatic I/R injury

**FIGURE 4:**
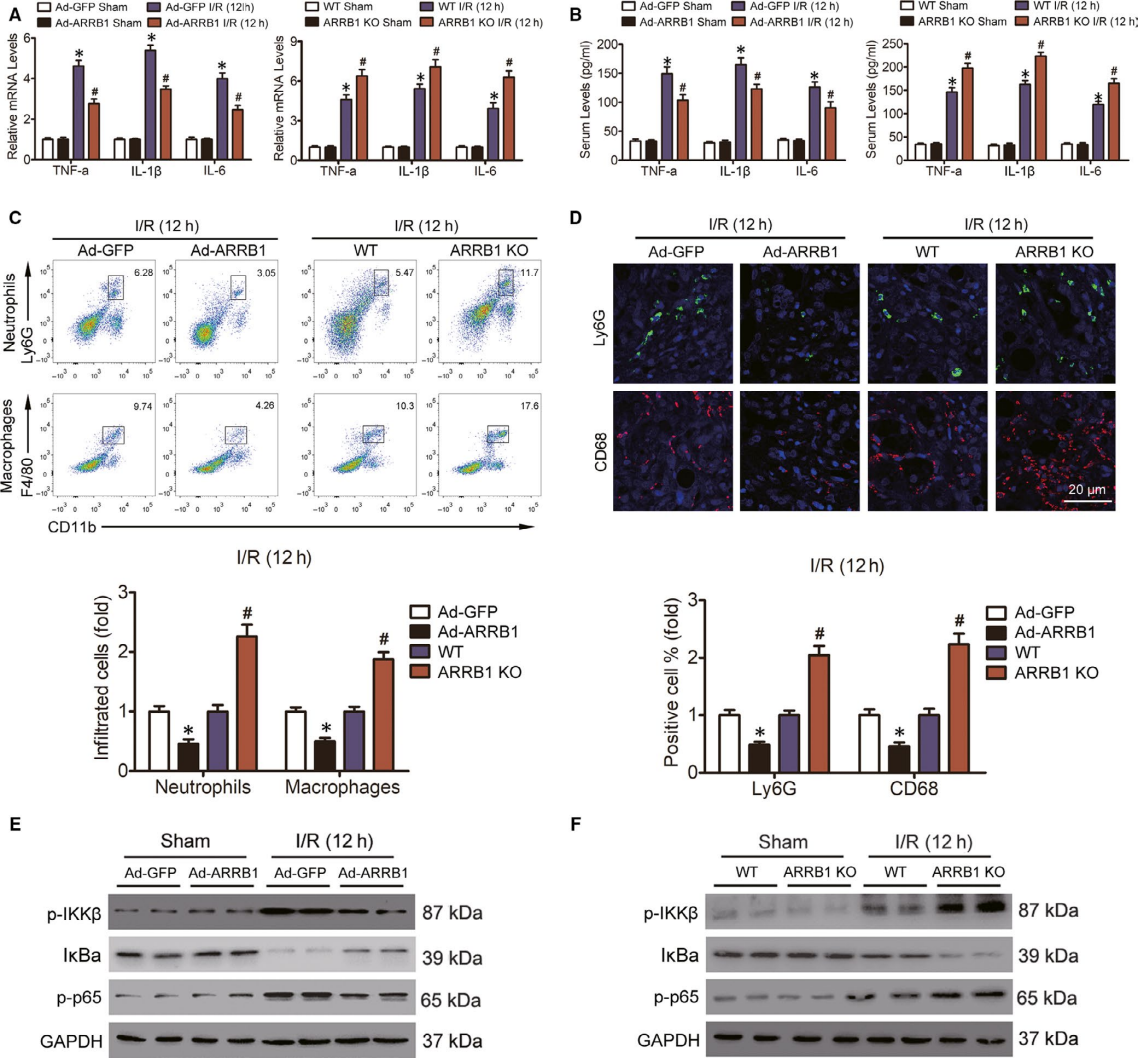
ARRB1 restrains inflammatory response in livers during hepatic I/R injury. A, The mRNA levels of pro‐inflammatory cytokines in mouse liver and the production of them in the serum (B) of mice were measured 12 h post‐reperfusion (n = 5‐6 per group). C, Flow cytometry analysis was conducted to examine the accumulation of neutrophils and macrophages in livers from mice subjected to 12 h post‐reperfusion (n = 5‐6 per group). D, Representative immunofluorescence staining of Ly6G‐positive cells (green) and CD68‐positive cells (red) in the livers of Ad‐GFP and Ad‐ARRB1, WT and ARRB1 KO mice 12 h after I/R injury (magnification, ×400, n = 5‐6 per group). E and F, Immunoblotting analysis of the activation of NF‐κB signalling in livers from these mice 12 h post‐reperfusion. GAPDH served as the loading control. For A and B, *P < .05 compared with the sham group, ^#^ *P* < .05 compared with the corresponding Ad‐GFP or WT groups; for C and D, *P < .05 indicates Ad‐ARRB1 compared with Ad‐GFP, ^#^ *P* < .05 indicates ARRB1 KO compared with WT. All data are presented as the mean ± SD; significance was determined by one‐way ANOVA

Sterile inflammation is the main characteristic of liver I/R injury, which occurs immediately after ischaemic stimulation and continues throughout the process of I/R injury and can be boosted by cell death.^20^ Here, we proposed to determine the inflammatory response in Ad‐ARRB1 and ARRB1 KO mice after liver I/R injury and the corresponding situation in control mice. The up‐regulation and production of pro‐inflammatory cytokines during hepatic I/R injury could then in turn aggravate inflammation. Firstly, we examined whether ARRB1 in hepatocytes could regulate the expression and production of inflammation during hepatic I/R injury. We found that ARRB1 overexpression or deletion did not influence the basic inflammation status under sham conditions. However, post‐I/R injury significantly up‐regulated the production of pro‐inflammatory cytokines, including TNF‐a, IL‐1β and IL‐6, both the mRNA levels in liver scopes and the activities in mice serum, and overexpression of ARRB1 obviously alleviated their production, whereas deletion of ARRB1 resulted in an aggravation of this phenomenon (Figure 4A,B). Furthermore, the elevated and attenuated pro‐inflammatory cytokines production in Ad‐ARRB1 mice and ARRB1 KO mice was confirmed in the medium collected from primary hepatocyte cultures treated with H/R insult (Figure S3A,B). Next, flow cytometry assay was conducted to detect the inflammatory cell infiltration situation, as shown in Figure 4C, and the population of neutrophils and macrophages in the livers of Ad‐ARRB1 mice was decreased compared with the Ad‐GFP group, whereas knockdown of ARRB1 significantly increased the populations of neutrophils and macrophages in liver after hepatic I/R injury. In addition, the representative reduction and augment of inflammatory cell infiltration in the liver of Ad‐ARRB1 and ARRB1 KO mice post‐hepatic I/R injury was further confirmed by immunofluorescence assay (Figure 4D). The activation of NF‐κB signalling during liver I/R injury plays important role in adjusting the status of inflammation. In addition, ARRB1 has been reported to be involved in regulation of the NF‐κB signalling pathway in various disease.^21^, ^22^ Therefore, we hypothesized that the expression of ARRB1 in hepatocytes might participate in the activation of p65 during hepatic I/R injury. Here, as shown in Figure 4E, the phosphorylation status of IKKβ and p65 was decreased, whereas the total IκBa was increased upon ARRB1 overexpression in hepatocytes when treated with I/R challenge. By contrast, ARRB1 deletion obviously enhanced the activation of p65 during hepatic I/R injury (Figure 4F). Furthermore, this regulation of ARRB1 on the activation of p65 was also confirmed in vitro when hepatocytes were challenged with H/R insult (Figure S3C,D). Collectively, these data suggest that ARRB1 restrains inflammatory response in hepatic I/R injury.

### 3.5 ARRB1 modulates ASK1‐JNK/p38 signalling during hepatic I/R process

**FIGURE 5:**
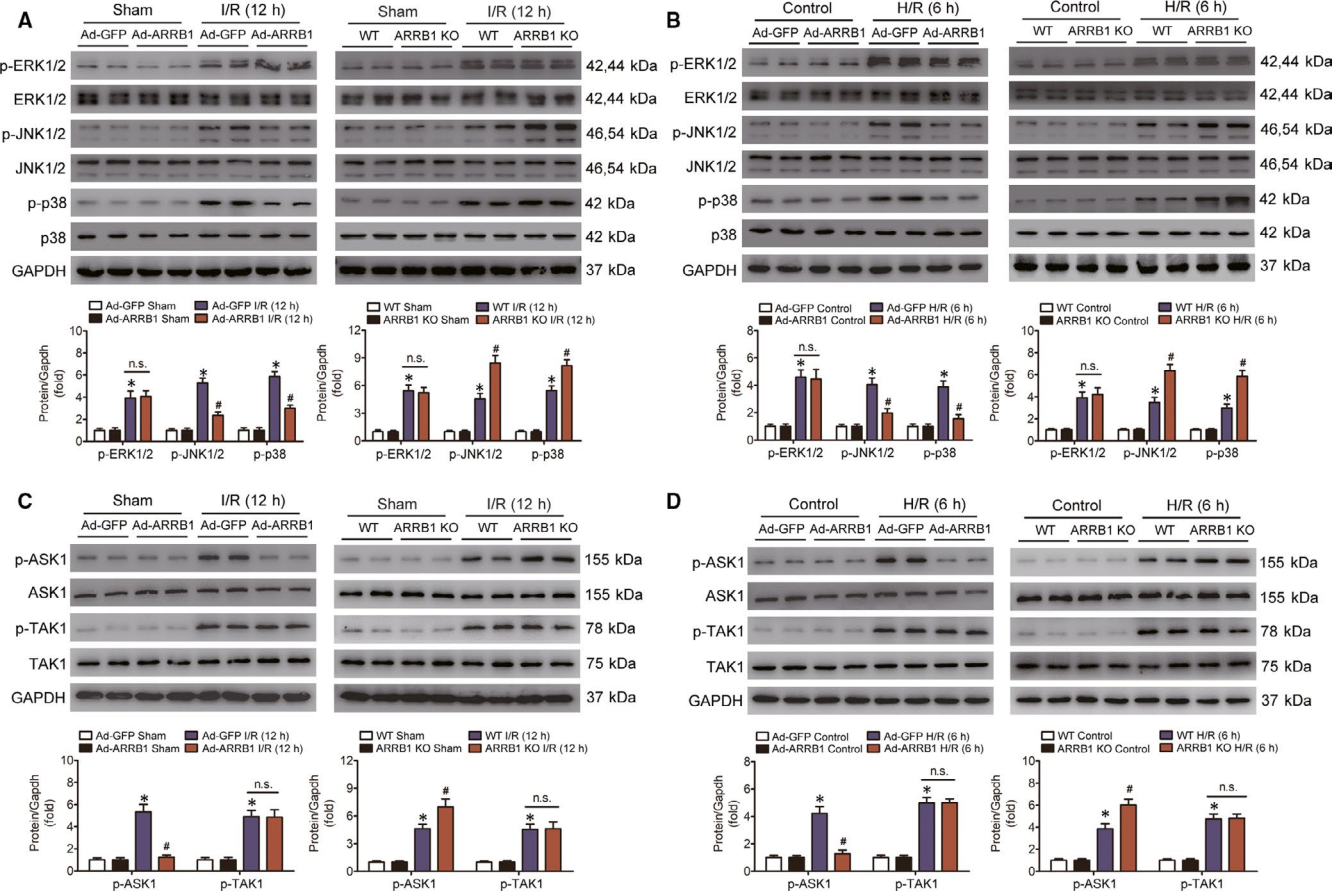
ARRB1 suppressed the activation of JNK/p‐38 via inhibiting ASK1 during hepatic I/R injury. A and B, Western blot analysis of the total and phosphorylation protein expression levels of the three classic MAPK proteins, that is, ERK1/2, JNK1/2 and p38, in mouse liver (A) and primary hepatocytes (B) after I/R or H/R challenge. C and D, Expression of the total and phosphorylated protein of ASK1 and TAK1 in mouse liver (C) and primary hepatocytes (D) obtained from Ad‐GFP, Ad‐ARRB1, WT and ARRB1 KO mice after I/R or H/R challenge. GAPDH served as the loading control. n = 5‐6 for the in vivo experiments; n = 3 independent experiments for the in vitro study. *P < .05 compared with sham or control groups; ^#^ *P* < .05 compared with the corresponding Ad‐GFP or WT I/R or H/R groups in A–D. All data are presented as the mean ± SD; significance was determined by one‐way ANOVA

The vital function of ARRB1 on liver I/R‐caused damage encourages us to determine the molecular mechanism underlying ARRB1 regulation of hepatic I/R injury. Studies have revealed that the mitogen‐activated protein kinase (MAPK) signalling pathway plays an important role in the regulation of both cell survival and inflammation during hepatic I/R injury.^23^ Researches investigating the regulation function of ARRB1 on MAPK signalling pathway have got a contrary conclusion in various diseases,^24^, ^25^ but whether and how it regulates MAPK signalling pathway in hepatic I/R injury is still unknown. Thus, the expression levels of the phosphorylated form of three MAPK proteins, that is extracellular signal‐regulated kinase (ERK), c‐Jun‐N‐terminal kinase (JNK) and p38, were detected. As shown in Figure 5A, all three kinases were activated upon I/R insult, and overexpression of ARRB1 significantly inhibited the expression of p‐JNK1/2 and p‐p38, whereas deletion of ARRB1 enhanced the activation of both kinases compared with the control groups. In both conditions, the phosphorylation status of ERK1/2 was not changed. In addition, these results were also confirmed in the primary hepatocyte cultures challenged with H/R insult (Figure 5B).

Studies have revealed that the upstream kinases ASK1 and TAK1 are the vital regulators of MAPK signalling during hepatic I/R injury.^26^, ^27^ Here, to further investigate how ARRB1 inhibited the JNK1/2 and p38 signalling upon liver I/R challenge, we purposed to detect whether ARRB1 was involved in modulating these two important kinases. Obviously, as shown in Figure 5C, both ASK1 and TAK1 were activated upon hepatic I/R insult, and overexpression or knockout of ARRB1 did not influence the basal phosphorylation status of both ASK1 and TAK1 under sham condition. ARRB1 overexpression significantly inhibited the phosphorylation level of ASK1, whereas knockout of ARRB1 enhanced the level of phosphorylated ASK1 when treated with liver I/R surgery. However, the phosphorylation level of TAK1 was not changed when ARRB1 was overexpressed or knockout in respond to liver I/R operation. Also, this effect of ARRB1 on ASK1 was obtained in the in vitro H/R treatment (Figure 5D). These findings indicate that ARRB1 may modulate JNK1/2 and p38 signalling through restraining the activation of ASK1.

### 3.6 ARRB1 interacts with ASK1 and then inhibits its activation through antagonizing TRAF6‐mediated Lysine6‐linked polyubiquitination in hepatocytes during hepatic I/R injury

**FIGURE 6:**
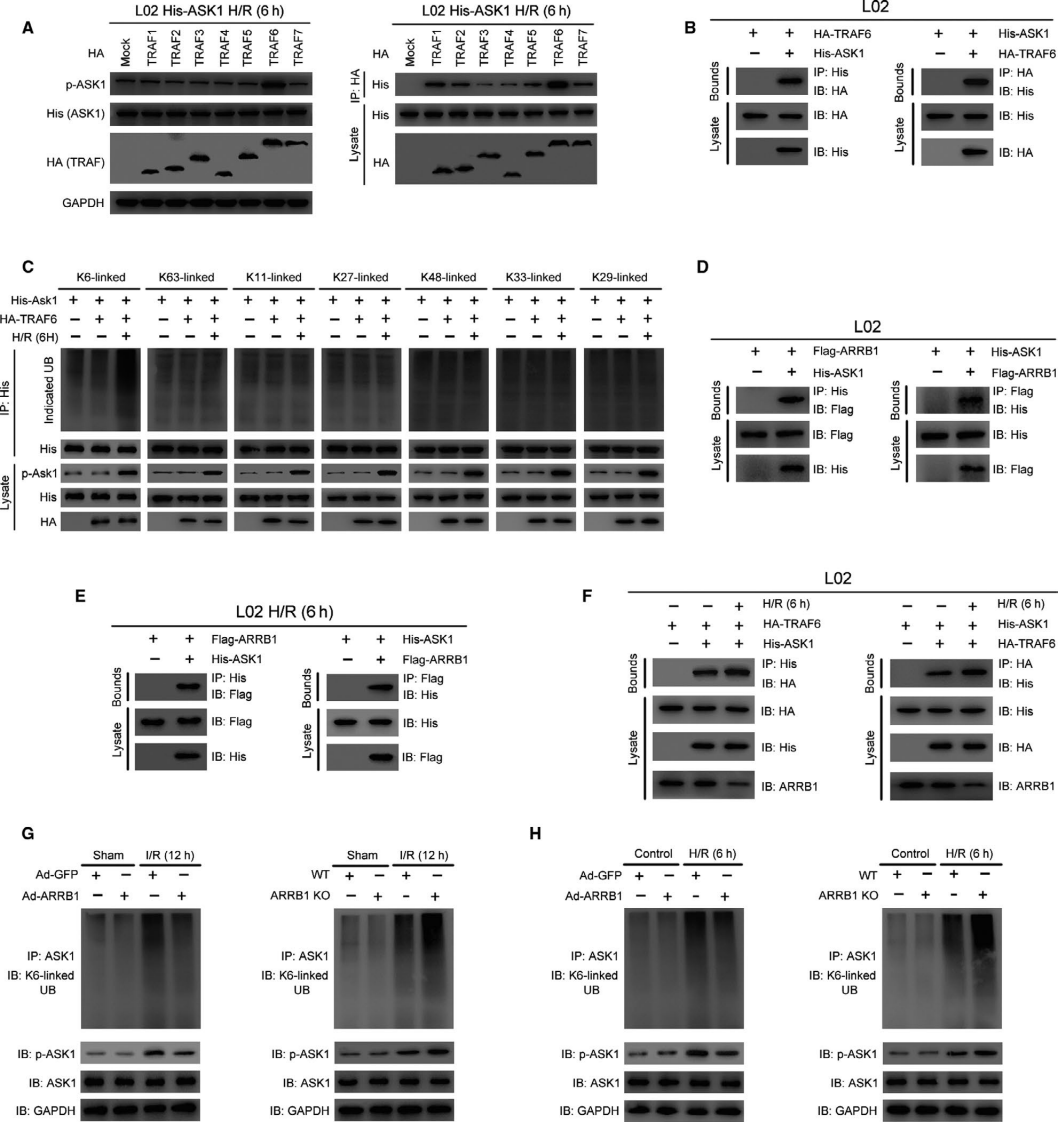
ARRB1 interacts with ASK1 and then antagonizes its TRAF6‐mediated K6‐linked polyubiquitination, thus inhibiting its activation in hepatocytes during hepatic I/R injury. A, Immunoblotting detection of phosphorylated ASK1 protein (left panel) and co‐IP assay for the interaction between His‐ASK1 and HA‐TRAF proteins (right panel) in L02 hepatocytes stimulated with H/R for 6 h. B, His‐tagged ASK1 and HA‐tagged TRAF6 plasmids were cotransfected into L02 hepatocytes. Anti‐His antibody (left panel) and anti‐HA antibody (right panel) were used for immunoprecipitation. C, Immunoprecipitation analysis of indicated ubiquitination types of ASK1 in L02 hepatocytes transfected with HA‐TRAF6 or empty vector in respond to H/R insult or not. D, IP analysis was conducted to detect the binding association between ASK1 and ARRB1 in L02 hepatocytes under normal condition. His‐tagged ASK1 and Flag‐tagged ARRB1 plasmids were cotransfected into L02 hepatocytes. Anti‐His antibody (left panel) and anti‐Flag antibody (right panel) were used for immunoprecipitation. E, IP analysis was conducted to detect the binding association between ASK1 and ARRB1 in L02 hepatocytes when treated with H/R challenge. Anti‐His antibody (left panel) and anti‐Flag antibody (right panel) were used for immunoprecipitation. F, IP analysis showing the expression level of ARRB1 and binding capability between ASK1 and TRAF6 in L02 hepatocytes under H/R challenge or not. Anti‐His antibody (left panel) and anti‐HA antibody (right panel) were used for immunoprecipitation. G and H, Lysates of liver lobes challenged with or without I/R surgery (G) or whole‐cell lysates of primary hepatocytes subjected to or not H/R insult (H) were subjected to immunoprecipitation with anti‐ASK1 antibody followed by immunoblotting with anti‐K6‐linked polyubiquitination antibody when ARRB1 was overexpressed or knockout

The TRAF family, constituting of seven members (TRAF1‐7), have been identified as ubiquitin ligases and then exert vital function in regulating signal transduction.^28^, ^29^ It has been demonstrated that multiple TRAF proteins are capable of binding ASK1 and modulating ASK1 activity, including TRAF2/3/6.^29^ In addition, among the seven members, TRAF6 has been demonstrated had the greatest capability to interact with ASK1 and then regulated the activation of ASK1 through promoting its Lysine 6‐linked polyubiquitination during the progression of non‐alcoholic steatohepatitis (NASH).^30^ However, whether this regulation exists in the process of liver I/R injury is still unknown. With the aim of identifying potential E3 ubiquitin ligase(s) involved in ASK1 activation during liver I/R injury, we investigated the function of TRAF proteins on phosphorylation level of ASK1 and the interaction intensity between TRAF proteins and ASK1 in human L02 hepatocytes under H/R challenge. As shown in Figure 6A, we found that among the TRAF proteins, TRAF6 had the greatest capability to interact with ASK1, and compared to other family members, only TRAF6 could greatly activate ASK1 during liver I/R injury. Next, we also confirmed the binding association between ASK1 and TRAF6 in L02 hepatocytes under normal condition (Figure 6B). And then, to explore the mechanism underlying TRAF6‐driven ASK1 activation during liver ischaemia‐reperfusion injury, we conducted a ubiquitination type screen in L02 cell treated with control treatment or H/R challenge and found that only K6 ubiquitination was specific for TRAF6‐mediated ASK1 ubiquitination and activation during hepatic I/R injury (Figure 6C).

Recently, ARRB1, but not ARRB2, was reported to bind with ASK1 upon treatment of melatonin in hepatocytes.^31^ Since our data have shown that ARRB1 could inhibit the activation of ASK1 during hepatic I/R injury, we proposed that ARRB1 might participate in the TRAF6‐mediated ASK1 activation in response to liver I/R injury. As shown in the Figure 6D,E, we have identified the interaction between ARRB1 and ASK1 in L02 cells, not only under normal condition but also under H/R challenge. Moreover, to investigate the endogenous binding of ARRB1 and ASK1/TRAF6, we used shRNA techniques to knockdown the expression of ARRB1 in L02 cells (Figure S3E), performed corresponding IP and Western blot assays and found that the binding ability of ASK1 and TRAF6 was significantly enhanced after ARRB1 knockdown in L02 cells (Figure S3F). In addition, to explore whether I/R influences their binding, we subjected corresponding L02 cells to H/R insult and followed IP and Western blot assays and found that ARRB1 expression was obviously down‐regulated in hepatocytes after H/R stimulation, followed by significantly enhanced binding ability of ASK1 and TRAF6 (Figure 6F). Next, we examined the K6‐linked polyubiquitination status of ASK1 in hepatocytes upon ARRB1 overexpression or knockout when challenged with liver I/R surgery or H/R treatment. As shown in Figure 6G, the K6‐linked polyubiquitination of ASK1 was inhibited when ARRB1 was overexpressed and the level of phosphorylated but not total ASK1 was decreased, whereas ARRB1 knockout strengthened TRAF6‐mediated K6‐linked polyubiquitination of ASK1 and increased its phosphorylation level in hepatocytes upon hepatic I/R challenge. In addition, this phenomenon was gotten in primary hepatocytes in vitro when treated with H/R insult (Figure 6H). Taken together, we confirmed that ARRB1 regulates the activation of ASK1 through antagonizing TRAF6‐mediated K6‐linked polyubiquitination during hepatic I/R injury.

### 3.7 ASK1 is the target for the protective effect of ARRB1 in liver I/R injury

**FIGURE 7:**
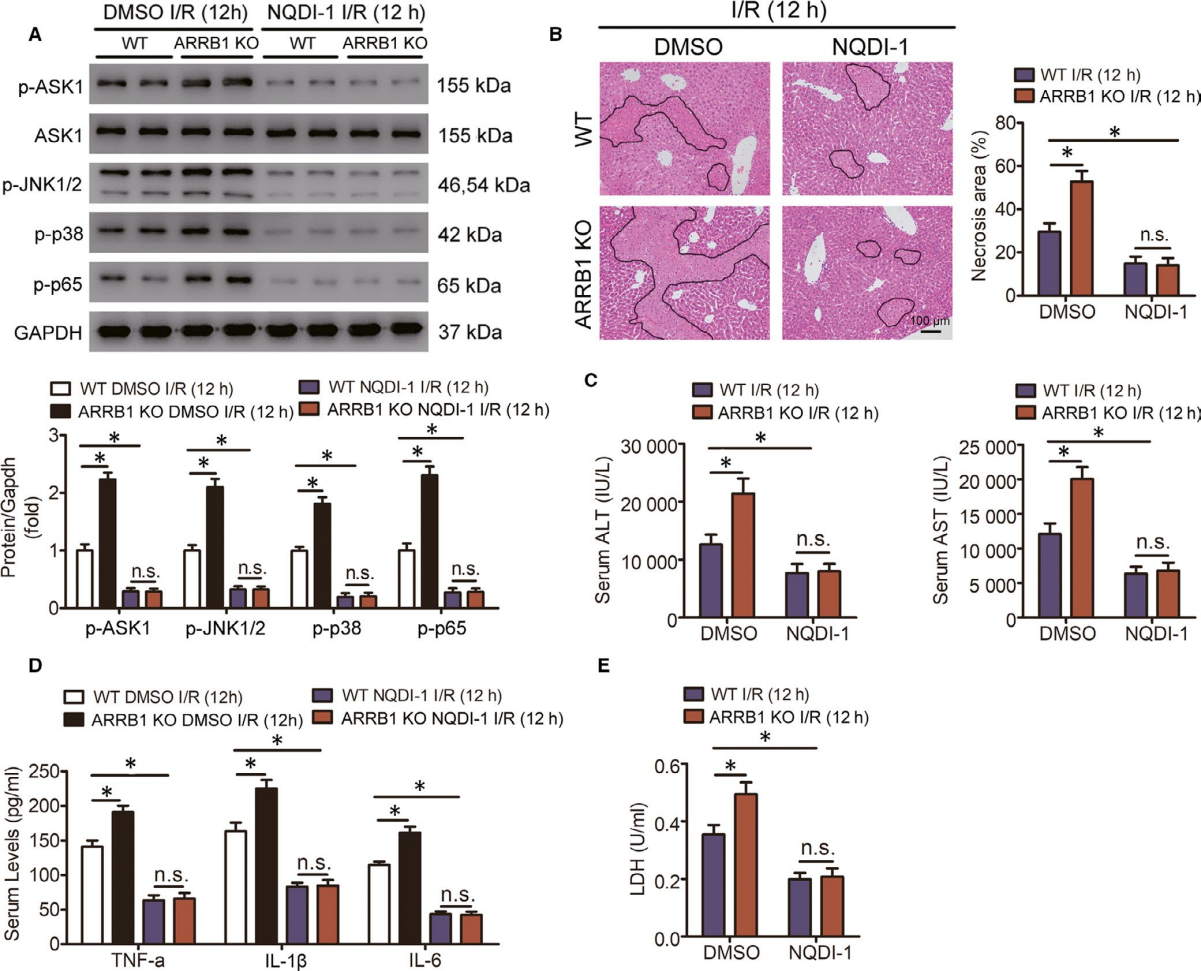
ASK1 mediates the function of ARRB1 during liver I/R injury. ASK1 inhibitor NQDI‐1 or DMSO (control group) was injected with ARRB1 KO and WT mice before I/R operation. A, Western blot was conducted to detect the expression levels of p‐ASK1, ASK1, p‐JNK1/2, p‐p38 and p‐p65 in ARRB1 KO and WT mice pretreated with NQDI‐1 or DMSO after liver I/R injury for 12 h. B, Representative images and analysis of histological H&E staining showing necrotic areas in livers from ARRB1 KO or WT mice pretreated with NQDI‐1 or DMSO and subjected to hepatic I/R for 12 h (magnification, ×200, n = 5‐6 per group). C, The activities of serum AST and ALT and (D) pro‐inflammatory cytokines in the indicated group (n = 5‐6 per group). E, LDH release of primary hepatocytes isolated from ARRB1 KO and WT mice injected with NQDI‐1 or DMSO. *P < .05; all data are presented as mean ± SD; significance determined by one‐way ANOVA

To further explore whether the protective effect of ARRB1 in hepatic I/R injury was achieved through regulating the activation of ASK1, we next established recuse experiments through inhibiting the activation of ASK1. We used NQDI‐1 to inhibit the activation of ASK1 and DMSO served as the control. As shown in Figure 7A, pretreatment with NQDI‐1 before I/R operation significantly suppressed ASK1 activation and its downstream signalling pathways, including JNK1/2, p38 and p65 in liver. Obviously, inhibition of ASK1 significantly defend liver from I/R injury and reversed the liver damage caused by knockout of ARRB1, shown by less necrosis area by histological analysis (Figure 7B), reduced serum ALT and AST activities (Figure 7C) and decreased production of pro‐inflammatory cytokines (Figure 7D). In addition, treatment with NQDI‐1 obviously reduced the production of LDH in primary hepatocytes and normalized the damage caused by ARRB1 deficiency (Figure 7E). Altogether, we suggest that inhibition of ASK1 remarkably reversed the disruptive effect result from ARRB1 deficiency in liver I/R injury, and ASK1 was the target for the protective effect of ARRB1 in liver I/R injury.

## 4 DISCUSSION

Since the concept of reperfusion injury was first put forward in 1960s, the research on solving this clinicopathological problem has been greatly developed in the past few decades.^32^, ^33^ Nevertheless, in clinical practice, only preconditioning is a promising strategy with limited scope of application and beneficial effects so far.^3^ In our present study, on account of genetic alteration strategies and abundant functional experiments, we proposed that ARRB1 might serve as a promising therapeutic target for hepatic I/R injury. Evidently, overexpression of ARRB1 in hepatocyte significantly attenuated cell death and inflammatory response resulting from hepatic I/R insult or hepatocyte H/R challenge, whereas deletion of ARRB1 obviously deteriorates the damage induced by I/R or H/R. Furthermore, we also elucidated that ARRB1 brought protection function during hepatic I/R injury was mainly dependent on its interaction with ASK1, which antagonized TRAF6‐mediated K6‐linked polyubiquitination and then blocks the activation of ASK1 and its downstream signalling.

As is known, the occurrence of I/R in the liver is transient, and the subsequent pathological development is dynamic. Elements which are sensitive to the occurrence and response of the entire process may be central triggers and drivers of hepatic I/R injury. Here, we proposed that ARRB1 might be such a factor. And what's more, the obvious alteration of the protein and mRNA levels of ARRB1 gotten from the mice hepatic I/R surgery indicates its potential clinical application value. It has been revealed that expression of ARRB1 in hepatocyte could enhance hepatocellular carcinogenesis through aggravating inflammation‐mediate signalling pathway. Moreover, deficiency of ARRB1 caused increased mass accumulation and decreased whole body insulin sensitivity in mice fed a high‐fat diet.^34^ And our recently study demonstrated that ARRB1 could act as a vital regulator in the development of NASH via facilitating GDF15's translocation to the Golgi apparatus and subsequent maturation.^17^ In addition, deficiency of ARRB1 impairs inflammatory response thus ameliorating collagen‐induced arthritis.^35^ Furthermore, ARRB1 could function as a protective factor in cerebral ischaemia through suppressing I/R‐induced neuronal apoptosis and necrosis.^12^ Similarly, our data clearly demonstrate that ARRB1 could attenuate cell death and inflammatory response in hepatic I/R or H/R insult, which expand our conclusion that targeting ARRB1 may facilitate clinical therapy for hepatic I/R injury.

MAPK and NF‐κB signalling have been recognized to play profound biological functions, including cell death and inflammation, and are widely established to be involved in the progression of hepatic I/R injury.^36^, ^37^ ARRB1 has been validated to regulate other signalling pathways besides terminating G protein‐coupled receptor signalling, including MAPK and NF‐κB signalling pathways. However, conflicting roles of ARRB1 in these two signalling have been reported in different diseases. For instance, ARRB1 may act as inhibitor of the activation of ERK upon the angiotensin receptor was activated,^24^ whereas ARRB1 is responsible for G protein‐independent ERK1/2 activation by the beta2 adrenergic receptor.^25^ ARRB1 has been reported to involve in attenuation of NF‐κB‐dependent transcription in response to GPCR or cytokine stimulation by interacting with and stabilizing CHUK. However, research in ovarian cancer suggested that ARRB1 is required for endothelin‐1‐induced NF‐κB activation.^38^ In our study, Ad‐ARRB1 and ARRB1 KO mice did not show altered MAPK or NF‐κB signalling at the basal level. However, Ad‐ARRB1 mice did exist attenuated hepatic I/R injury‐induced MAPK and NF‐κB signalling activation, while deletion of ARRB1 enhanced their activation.

The classical function of β‐arrestins is achieved by mediating the internalization and degradation of GPCR family molecules. Here, through literature retrieval, we found that so far, studies on β‐arrestins in ischaemia‐reperfusion injury revealed that its function was mainly achieved through non‐classical GPCR‐independent pathways. For example, in heart, the expression of ARRB2, but not ARRB1, is up‐regulated in cardiac ischaemia‐reperfusion injury, which then induces cardiomyocyte death by interacting with the p85 subunit of PI3K and negatively regulates the formation of p85‐PI3K/CaV3 survival complex.^13^ In brain, overexpression or activation of ARRB1, but not ARRB2, exerts neuroprotection through coordination of BECN‐1‐dependent autophagy in cerebral ischaemia.^12^ Recently, ARRB1, but not ARRB2, was reported to bind with ASK1 upon treatment of melatonin in hepatocytes during the pathology of NASH.^31^ ASK1 has been identified as a vital factor in hepatic I/R injury via activating its downstream MAPK or NF‐κB signalling.^26^ In our present study, we fortunately found that ARRB1 was involved in the regulation of ASK1‐JNK/p38 pathways when detecting the classical pathways for liver ischaemia‐reperfusion injury. Thus, in our present study, we mainly focused on its underlying regulatory function on ASK1 during liver ischaemia‐reperfusion injury. In addition, TRAF6 has been demonstrated had the greatest capability to interact with ASK1 and then regulated the activation of ASK1 through promoting its Lysine 6‐linked polyubiquitination during the progression of non‐alcoholic steatohepatitis (NASH).^30^ Here, through IP, Western blot and ubiquitination type screen assays, we confirmed that among the TRAF proteins, TRAF6 had the greatest capability to interact with ASK1, and compared to other family members, only TRAF6 could greatly activate ASK1 by enhancing Lys6‐linked ubiquitination in hepatocytes during hepatic I/R injury. Also, the binding partner between ASK1 and ARRB1 was validated in hepatocytes, and ARRB1 inhibited the activation of ASK1 by antagonizing TRAF6‐mediated Lysine 6‐linked polyubiquitination during hepatic I/R injury. In addition, in *vivo* rescue experiments revealed that inhibition of ASK1 remarkably reversed the disruptive effect result from ARRB1 deficiency in liver I/R injury. These observations together suggested that ASK1 is a vital target for ARRB1, and suppression the activation of ASK1 is important to ARRB1‐mediated protective effect on liver I/R injury.

In conclusion, our present study has identified ARRB1 as a novel protective factor of hepatic I/R injury by interacting with ASK1 and then inhibiting its TRAF6‐mediated Lysine 6‐linked polyubiquitination, thus restraining the activation of ASK1 and its downstream signalling. ARRB1 overexpression in hepatocyte significantly attenuates cell death and inflammation response during hepatic I/R injury. Furthermore, inhibition of ASK1 significant normalized the liver I/R damage aggravated by loss of ARRB1. Thus, targeting ARRB1 or modulating the ARRB1‐ASK1 interaction may provide a promising therapeutic approach for liver I/R injury.

## CONFLICT OF INTEREST

All authors state that they have no conflicts of interest.

## AUTHOR CONTRIBUTIONS

**Xiaoliang Xu:** Conceptualization (lead); Data curation (lead); Formal analysis (equal); Investigation (lead); Methodology (equal); Resources (equal); Software (equal); Writing‐original draft (lead); Writing‐review & editing (lead). **Zechuan Zhang:** Data curation (equal); Formal analysis (equal); Investigation (equal); Methodology (equal); Software (equal); Writing‐review & editing (equal). **Yijun Lu:** Formal analysis (equal); Investigation (equal); Software (equal); Validation (equal); Visualization (equal); Writing‐review & editing (equal). **Qikai Sun:** Investigation (equal); Methodology (equal); Software (equal). **Yang Liu:** Investigation (equal); Methodology (equal); Visualization (equal). **Qiaoyu Liu:** Investigation (equal); Methodology (equal). **Wenfang Tian:** Investigation (equal); Software (equal); Validation (equal). **Yin Yin:** Software (equal); Validation (equal); Visualization (equal). **Hailong Yu:** Resources (equal); Software (equal); Visualization (equal). **Beicheng Sun:** Conceptualization (equal); Funding acquisition (lead); Project administration (lead); Writing‐review & editing (equal).

## Supporting information

Supplementary Material

## Data Availability

The data that support the findings of this study are available from the corresponding author upon reasonable request.
