# Supplementary material for: ARRB1 ameliorates liver ischaemia/reperfusion injury via antagonizing TRAF6‐mediated Lysine 6‐linked polyubiquitination of ASK1 in hepatocytes
Source: J Cell Mol Med. 2020 May 23;24(14):7814–28. doi: 10.1111/jcmm.15412 (PMC7348167; doi:10.1111/jcmm.15412)
Supplement: Supplementary file 1 — Supplementary Material [file JCMM-24-7814-s001.docx]

**ARRB1 ameliorates liver ischemia/reperfusion injury via antagonizing TRAF6-mediated Lysine 6-linked polyubiquitination of ASK1 in hepatocytes**

Xiaoliang Xu,^1,2,*^ Zechuan Zhang,^2,*^ Yijun Lu,^2,*^ Qikai Sun,^2^ Yang Liu,^2^ Qiaoyu Liu,^2^ Wenfang Tian,^2^ Yin Yin,^2^ Hailong Yu,^2^ Beicheng Sun^1,2,#^

^1^School of Medicine, Southeast University, Nanjing, China; ^2^Department of Hepatobiliary Surgery, The Affiliated Drum Tower Hospital of Nanjing University Medical School, Nanjing, China.

^*^These authors contributed equally to this work.

^#^Correspondence: School of Medicine, Southeast University, Nanjing 210008, Jiangsu Province, China; Department of Hepatobiliary Surgery, Nanjing Drum Tower Hospital, Clinical College of Nanjing Medical University, 321 Zhongshan Rd., Nanjing 210008, Jiangsu Province, China. E-mail: [sunbc@nju.edu.cn](mailto:sunbc@nju.edu.cn).

**Supplementary Figures**

**
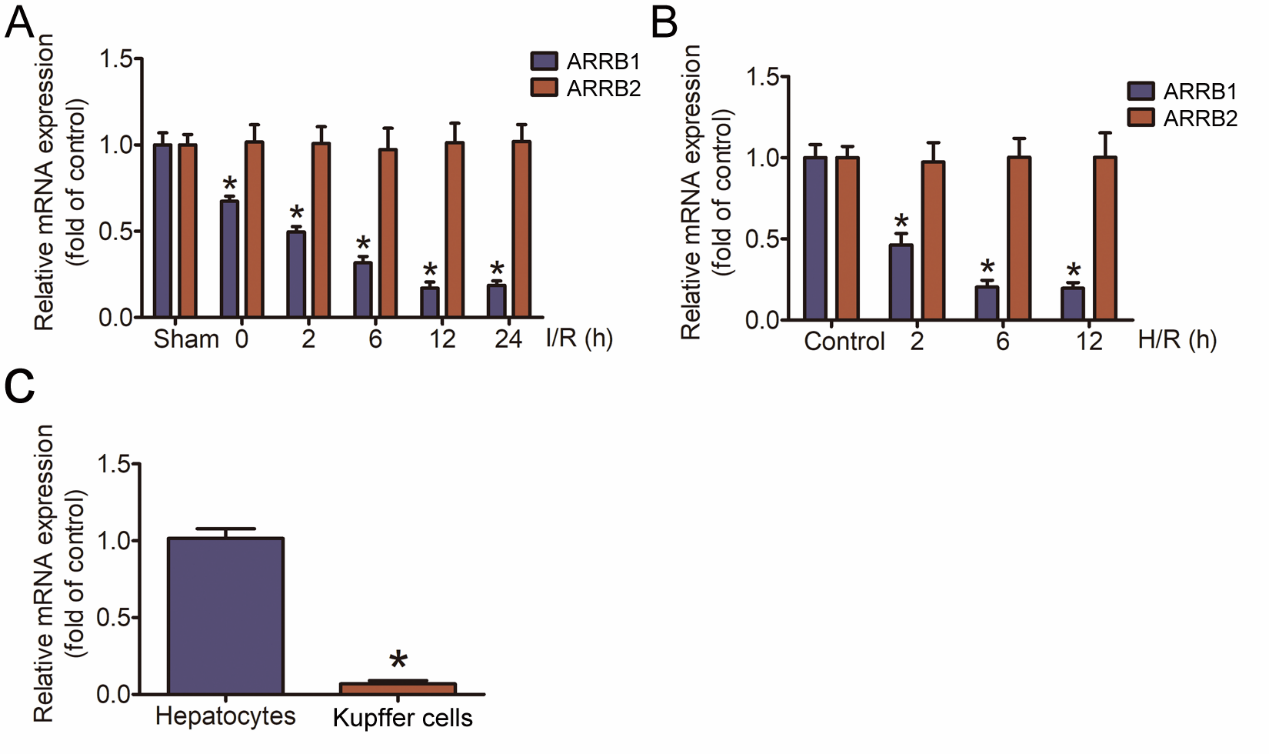
**

**Supplementary Figure 1. The mRNA expression levels of ARRB1 and ARRB2 during hepatic I/R or H/R injury.** (A) RT-qPCR analysis of the mRNA levels of ARRB1 and ARRB2 in livers from mice subjected to sham treatment or ischemia 1 hour followed by reperfusion for indicated times (n = 5-6 per group, **P* < 0.05 compared with sham group). (B) The mRNA expression levels of ARRB1 and ARRB2 in primary hepatocytes after sham or H/R treatment (Representative of three independent experiments, **P* < 0.05 compared with the control group). (C) The mRNA expression level of ARRB1 in mouse hepatocytes and Kupffer cells was examined by RT-qPCR (n = 4 per group, **P* < 0.05 compared with hepatocytes). GAPDH served as the loading control. All data were presented as the mean ± SD; significance determined by Student’s two-tailed *t* test.

**
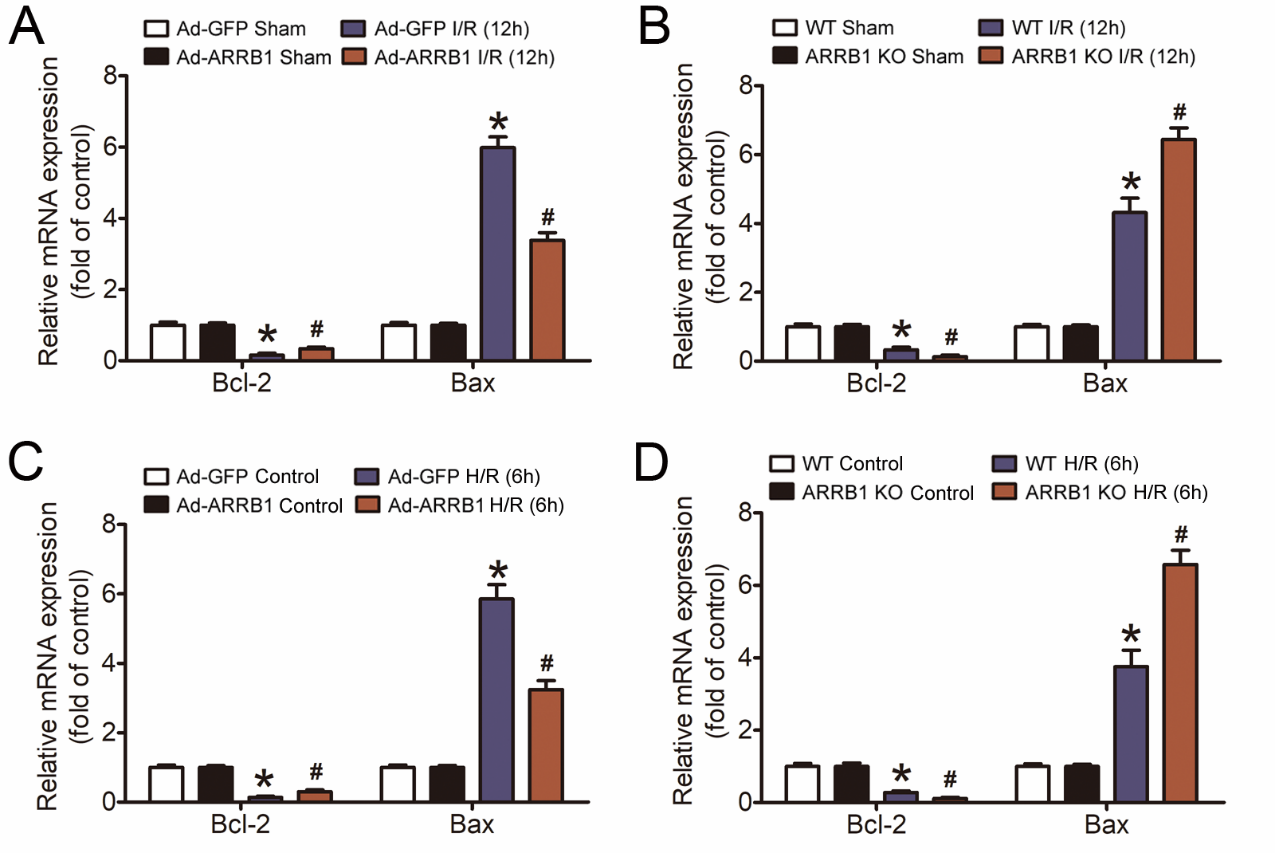
**

**Supplementary Figure 2. The mRNA expression levels of BCL-2 and BAX during hepatic I/R or H/R injury.** (A and B) mRNA levels of cell death-related factors in liver lobes from the indicated groups at 12 hours after I/R insult (n = 5-6 per group). (C and D) Cell death-associated gene mRNA expression in the primary hepatocytes of different groups at 6 hours post-H/R treatment. n = 3 independent experiments for the in vitro study. GAPDH served as the loading control. **P* < 0.05 compared with sham or control groups; #*P* < 0.05 compared with corresponding Ad-GFP or WT I/R or H/R groups. All data were presented as the mean ± SD; significance determined by one-way ANOVA.

**
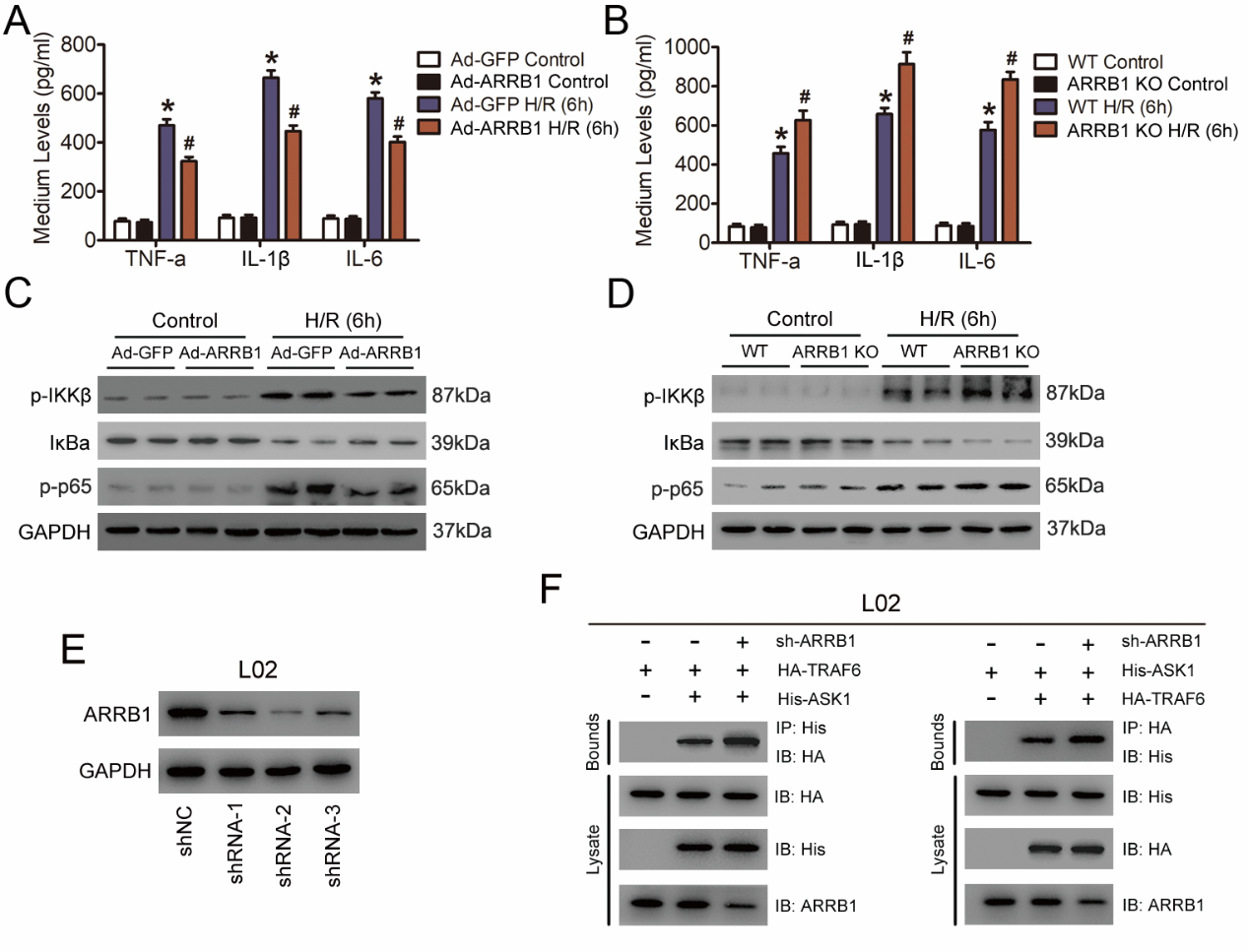
**

**Supplementary Figure 3. The production of pro-inflammatory cytokines from hepatocytes after hepatic H/R insult.** (A and B) The expression levels of pro-inflammatory cytokines from hepatocytes challenged with or without H/R insult were detected by ELISA (n = 3 independent experiments, **P* < 0.05 compared with the control group; #*P* < 0.05 compared with the corresponding Ad-GFP or WT groups). (C and D) The activation of NF-κB signaling in the primary hepatocytes during in vitro H/R challenge was detected by western blot. (E) shRNAs targeting ARRB1 were constructed and transfected into L02 hepatocytes, and the knock-down efficiency was detected by western blotting. (F) IP analysis showing the binding capability between ASK1 and TRAF6 in L02 hepatocytes when ARRB1 was knockdown or not. Anti-His antibody (left panel) and Anti-HA antibody (right panel) were used for immunoprecipitation. All data were presented as the mean ± SD; significance determined by one-way ANOVA.

**Supplementary Tables**

**Supplementary Table 1. Primers for RT-qPCR Analysis**

| **Gene** | **Sequence 5′−3′** |  |
| --- | --- | --- |
| ARRB1 | F: AGGCAAGCCCCAATGGAAAG | |
|  | R: AGTGTCACGTAGACTCGCCTT | |
| ARRB2 | F: AGTCGAGCCCTAACTGCAAG | |
|  | R: ACGAACACTTTCCGGTCCTTC | |
| IL-1β | F: GAAATGCCACCTTTTGACAGTG | |
|  | R: TGGATGCTCTCATCAGGACAG | |
| TNF-α | F: CAGGCGGTGCCTATGTCTC | |
|  | R: CGATCACCCCGAAGTTCAGTAG | |
| IL-6 | F: CTGCAAGAGACTTCCATCCAG | |
|  | R: AGTGGTATAGACAGGTCTGTTGG | |
| BCL-2 | F: GCTACCGTCGTGACTTCGC | |
|  | R: CCCCACCGAACTCAAAGAAGG | |
| BAX | F: AGACAGGGGCCTTTTTGCTAC | |
|  | R: AATTCGCCGGAGACACTCG | |
| GAPDH | F: CGTCCCGTAGACAAAATGGT | |
|  | R: TTGATGGCAACAATCTCCAC | |

**Supplementary Table 2. Antibodies for the Western Blot Analysis**

| **Name** | **Supplier** | **Cat No.** |
| --- | --- | --- |
| ARRB1 | Abcam | ab32099 |
| ARRB2 | Abcam | ab31294 |
| BCL-2 | Cell Signaling Technology | 3498 |
| BAX | Cell Signaling Technology | 2772 |
| p-p38 | Cell Signaling Technology | 4511 |
| p38 | Cell Signaling Technology | 9212 |
| p-JNK | Cell Signaling Technology | 4668 |
| JNK | Cell Signaling Technology | 9252 |
| p-ERK | Cell Signaling Technology | 4370 |
| ERK | Cell Signaling Technology | 4695 |
| p-IKKβ | Abcam | ab194519 |
| IKKβ | Abcam | ab124957 |
| p-IκBα | Abcam | ab133426 |
| IκBα | Abcam | ab32518 |
| p-p65 | Abcam | ab86299 |
| p65 | Abcam | ab16502 |
| p-ASK1 | Cell Signaling Technology | 3765 |
| ASK1 | Cell Signaling Technology | 8662 |
| p-TAK1 | Cell Signaling Technology | 9339 |
| TAK1 | Cell Signaling Technology | 5206 |
| GAPDH | Abcam | ab8245 |
| Flag | Medical & Biological Laboratories | M185 |
| HA | Cell Signaling Technology | 3724 |
| His | Cell Signaling Technology | 12698 |
